# Exposure to the Functional Bacterial Amyloid Protein Curli Enhances Alpha-Synuclein Aggregation in Aged Fischer 344 Rats and *Caenorhabditis elegans*

**DOI:** 10.1038/srep34477

**Published:** 2016-10-06

**Authors:** Shu G. Chen, Vilius Stribinskis, Madhavi J. Rane, Donald R. Demuth, Evelyne Gozal, Andrew M. Roberts, Rekha Jagadapillai, Ruolan Liu, Kyonghwan Choe, Bhooma Shivakumar, Francheska Son, Shunying Jin, Richard Kerber, Anthony Adame, Eliezer Masliah, Robert P. Friedland

**Affiliations:** 1 Dept. of Pathology, Case Western Reserve University, Cleveland, Ohio, USA; 2 Brown Cancer Center, University of Louisville School of Medicine, Louisville, Kentucky, USA; 3 Dept. of Medicine, University of Louisville School of Medicine, Louisville, Kentucky, USA; 4 Dept. of Oral Immunology and Infectious Diseases, University of Louisville School of Dentistry, Louisville, Kentucky, USA; 5 Dept. of Pediatrics, University of Louisville School of Medicine, Louisville, Kentucky, USA; 6 Dept. of Physiology, University of Louisville School of Medicine, Louisville, Kentucky, USA; 7 Dept. of Neurology, University of Louisville School of Medicine, Louisville, Kentucky, USA; 8 Dept. of Epidemiology and Population Health, University of Louisville School of Public Health, Louisville, Kentucky, USA; 9 Laboratory of Experimental Neuropathology, University of California, San Diego, California, USA.

## Abstract

Misfolded alpha-synuclein (AS) and other neurodegenerative disorder proteins display prion-like transmission of protein aggregation. Factors responsible for the initiation of AS aggregation are unknown. To evaluate the role of amyloid proteins made by the microbiota we exposed aged rats and transgenic *C*. *elegans* to *E*. *coli* producing the extracellular bacterial amyloid protein curli. Rats exposed to curli-producing bacteria displayed increased neuronal AS deposition in both gut and brain and enhanced microgliosis and astrogliosis compared to rats exposed to either mutant bacteria unable to synthesize curli, or to vehicle alone. Animals exposed to curli producing bacteria also had more expression of TLR2, IL-6 and TNF in the brain than the other two groups. There were no differences among the rat groups in survival, body weight, inflammation in the mouth, retina, kidneys or gut epithelia, and circulating cytokine levels. AS-expressing *C*. *elegans* fed on curli-producing bacteria also had enhanced AS aggregation. These results suggest that bacterial amyloid functions as a trigger to initiate AS aggregation through cross-seeding and also primes responses of the innate immune system.

The neurodegenerative disorders Parkinson’s disease (PD), Alzheimer’s disease, fronto-temporal lobar degeneration, amyotrophic lateral sclerosis and related disorders all involve the neuronal accumulation of pathogenic aggregated amyloid proteins with prion-like features^1^. The proteins deposited in the brain in these disorders alpha synuclein (AS), Amyloid beta (AB), tau and TDP-43 are transmissible from cell to cell, from one region of the body to another and from humans to susceptible animal hosts^2^. However the agent or agents responsible for the initiation of the protein misfolding is unknown. Prusiner^3^ and others have suggested that the initial event is stochastic, but the possibility that it is initiated by environment amyloids requires consideration^4^,^5^. The early development of constipation and anosmia and the deposition of aggregated AS in intestinal neurons up to 20 years before diagnosis in PD suggests that the initiating factor comes from the gut (including mouth, nasal passages and intestines)^6^. This is also suggested by the early brain pathology in PD in the dorsal motor nucleus of the vagus in the medulla, which is a region affected early in scrapie^7^. Furthermore, inflammatory changes are found in neurodegenerative disorders with microgliosis, astrogliosis and activation of inflammatory cytokines and lymphocytes, but without leukocyte infiltration. The factors responsible for the variable expression of this sterile inflammation are also largely unknown.

Our gut presents the greatest opportunity for exposure to foreign organisms (our microbiota). It has been known since 2002 that the bacteria and fungi make functional extracellular amyloid proteins^8^. Bacterial amyloid proteins are highly conserved, are involved in biofilm formation and help the bacteria with invasion, host adhesion, and resistance to destruction^8^,^9^. The best studied bacterial amyloid protein is curli made by *Escherichia coli* and its key element, CsgA, has been found to contain amyloidogenic peptide repeat motifs shared by yeast and human prions^10^ and AS^11^. Cross–seeding, in which one amyloidogenic protein (curli, Tau, AB or AS, yeast prions, silk protein) causes another to adopt a beta-sheet structure has been documented^12^. There is excellent precedent for amyloid misfolding to be initiated through gut exposure (i.e., bovine spongiform encephalopathy, serum amyloid A amyloidosis^13^).

The role of the immune system in neurodegeneration has now been well documented in both histopathological as well as genetic studies^14^. It has recently been reported that circulating neutrophils contribute to AS pathology in transgenic mice^15^. Nevertheless, the role of immune changes in production of disease phenotypes are not well understood. Several groups have found that many older persons with AB deposits do not have dementia^16^, which can be a result of lessened immune responses in some individuals^17^.

It has been firmly established that commensal microbiota plays a vital role in the regulation of the immune system^18^. This is necessary because of the massive dose of foreign antigens present in the gut, requiring the immune system to be constantly vigilant of its contents. The first step of the immune response, the innate immune system, recognizes bacterial amyloid proteins using pathways involving TLR 2/1, NLRP3, CD14, NFkB and iNOS^19^. This is similar to the mechanisms involved in the immune system’s recognition of AS and AB. Toll like receptor 2 is important for inflammatory processes in PD and other neurodegenerative disorders and plays an important role in enhancing AS aggregation through inhibition of autophagy^20^. Consequently, priming of microglia and enhancement of related immune responses to AB, AS, and other misfolded proteins produced by exposure to bacterial amyloid can thus contribute to the development of dementia^16^.

We have hypothesized that amyloid proteins made by our commensal partners function to trigger misfolding of amyloid proteins such as AS through cross-seeding, and cause priming of the innate immune system thereby enhancing inflammatory responses to AS^4^. In order to evaluate this hypothesis, we exposed aged rats and transgenic nematodes expressing human AS to bacteria producing curli. Control animals were exposed to mutant bacteria lacking the operons required for curli production. We assessed AS accumulation and aggregation and inflammation using immunocytochemistry and related techniques. Our results in both rodents and nematodes illustrate the ability of bacterial amyloid to influence important aspects of neurodegenerative processes.

## Results

### Exposure to curli-producing bacteria enhances AS aggregation in rat brain and AS deposition in gut

**Figure 1:**
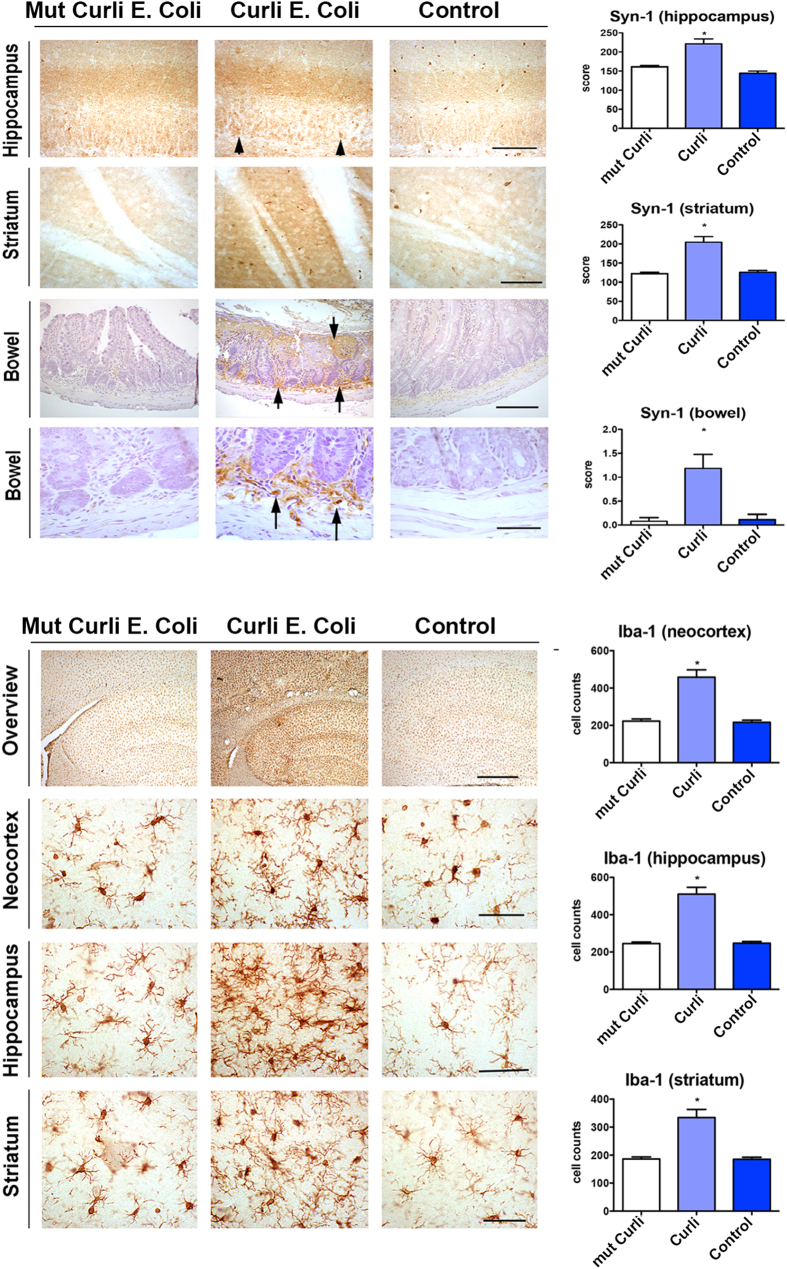
Rats exposed to bacteria expressing curli have enhanced AS deposition in hippocampus, striatum and gut neurons and increased growth of microglia. (**a**) Alpha-synuclein (Syn-1) immunohistochemical staining in gut, striatum and hippocampus. Gut neurons containing AS deposits are indicated with arrows, and hippocampal neurons containing AS are indicated with arrowheads. Data are for animals exposed to mutant bacteria unable to produce curli (Mut Curli *E*. *coli*), curli-producing wild type bacteria (Curli *E*. *coli*) and Control (exposed only to vehicle). For the striatum and hippocampus images the bars are 20 μm. For the bowel images the upper bar for the upper row of three bowel images is 250 μm and the lower bar for the lower row of three bowel images is 150 μm (higher magnification). Right panels represent quantification of the staining. *p < 0.05 when compared to control. (**b**) Iba1 (allograft inflammatory factor) staining of neocortex, hippocampus and striatum. The upper bar is 250 μm and the lower bars are 20 μm. The top images are lower magnification and the bottom three panels are higher magnification. *p < 0.05 when compared to control.

**Figure 2:**
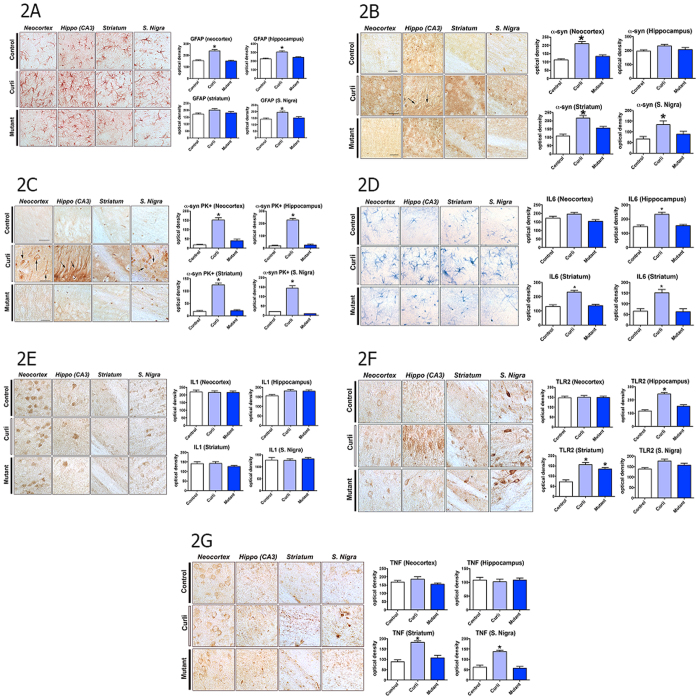
Rats exposed to bacteria expressing curli have increased astroglial growth, enhanced deposition of aggregated AS, and increased expression in brain of IL6, TLR2 and TNF. Immunohistochemical staining in neocortex, hippocampus, striatum and substantia nigra. Right panels represent quantification of the staining. Data are for animals exposed to bacteria unable to produce curli (Mut Curli *E*. *coli*), curli producing wild type bacteria (Curli *E coli)* and Control (exposed only to vehicle). Right panels represent quantification of the staining (**A**) Glial fibrillary acidic protein (GFAP) staining of neocortex, hippocampus (CA3) striatum and substantia nigra. *p < 0.05. (**B**) Alpha synuclein (α-syn) staining, without proteinase K treatment. *p < 0.05. (C****) Alpha synuclein (α-syn) staining following proteinase K treatment (PK+). Arrows represent deposits of proteinase K resistant AS in neurons. *p < 0.05. (**D**) Interleukin 6 (IL6) staining in neocortex, hippocampus (CA3) striatum and Substantia nigra. *p < 0.05. (**E**) Interleukin1 (IL1) staining in neocortex, hippocampus (CA3) striatum and Substantia nigra. (**F**) Toll like receptor 2 (TLR2) staining in neocortex, hippocampus (CA3) striatum and Substantia nigra. *p < 0.05. (**G**) Tissue necrosis factor (TNF) staining in neocortex, hippocampus (CA3) striatum and Substantia nigra. *p < 0.05. For images (**A**–**G**) the bars are 20 um.

Aged Fischer 344 rats have been described to have aggregated AS in the intestinal submucosal plexus^21^. We used these animals to evaluate the influence of exposure to bacteria producing amyloid proteins on AS deposition and aggregation in the gut as well as in the brain. Animals were exposed weekly via the oral route using an established method for bacterial exposure^22^. We found that rats exposed to wild-type bacteria producing the amyloid protein curli had enhanced AS deposition in gut ganglion cells (myenteric plexus and submucosa) (Fig. 1a, top panel). We also observed that rats exposed to wild-type bacteria producing curli had enhanced AS deposition in neurons in hippocampus (especially CA3) and striatum, as compared to rats exposed to mutant bacteria lacking the capacity to produce curli or to rats exposed only to vehicle (Fig. 1a). Deposits in neurons in the brain were proteinase K resistant, indicating the presence of aggregated AS (Fig. 2B,C). Quantitation showed that rats exposed to curli-producing bacteria had more AS deposition in the gut than the other 2 groups with an odds ratio of 2.5 (95% CI 4.4–239) demonstrating that exposed animals had a higher probability of having higher gut scores than unexposed animals (lower panel, Fig. 1a and Supplementary Figure 4). Alpha-synuclein aggregation was not related to length of exposure (2 or 3 months). AS deposits in the gut were proteinase K sensitive, suggesting the absence of AS aggregation in the gut.

### Exposure to curli-producing bacteria enhances immune responses in rat brain

AS as well as the bacterial amyloid protein curli are recognized by the innate immune system with involvement of similar pathways (TLR2, CD14, NFkB, iNOS). We hypothesized that exposure to bacterial amyloid would cause priming of immune cells to create enhanced immune responses in the brain of exposed animals. Therefore we assessed markers of neuroinflammation, including microgliosis, astrogliosis and the pro-inflammatory cytokines interleukin-6 (IL-6), interleukin-1 (IL-1) tissue necrosis factor (TNF) and TLR2. We observed that microgliosis (Iba-1, allograft inflammatory factor, Fig. 1b), as well as astrogliosis (GFAP, glial fibrillary acidic protein, Fig. 2A) in the striatum, hippocampus and neocortex was significantly higher in animals exposed to curli-producing bacteria as compared to the two control groups. Interleukin-6 (IL-6) expression was also higher in the hippocampus, stratum and substantia nigra of the animals exposed to curli than in the control groups (Fig. 2D). Toll like receptor 2 (TLR2) expression was also higher in the hippocampus and striatum in animals exposed to curli-producing bacteria as compared to the other 2 groups (Fig. 2F) and TNF expression was higher in striatum and substantia nigra of animals exposed to curli-producing bacteria than that in the other 2 groups (Fig. 2G). There were no significant differences in interleukin 1 expression (Fig. 2E). The results were not related to length of exposure.

### The phenotypes observed in curli-exposed rats were not related to illness, aging or cellular immune responses

In order to evaluate the possibility that the effects of exposure noted above were caused by illness induced by the bacteria we examined the relationship of the findings to body weight, evidence of inflammatory infiltrates, and survival. There were no significant differences among the 3 groups in terms of survival, body weight or cellular inflammation in oral tissues, kidneys, eyes, brain or gut (data not shown). We examined the retina in 12 eyes (4 eyes from each group) using standard histology and immunohistochemistry, as the eyes are located in proximity to oral tissues. The stratification of the retinal layers appeared similar across all eyes and groups. Frozen retinal sections (20 μm thickness) immunolabeled with GFAP (labeling astrocytes and activated Müller cells) and vimentin (labeling Müller cells) were similar across all eyes and groups (see Supplementary data).

### Serum cytokines and TLR-2

Because of our interest in systemic immune responses we evaluated serum levels of several cytokines and related molecules. Serum levels of IL-1β, IL-6, IL-10, and TNF-α were below detection levels. Serum INF-γ was detectable but there was no difference among three groups. Serum TLR2 levels were trending higher in the rats exposed to curli than the other two groups but the differences did not reach statistical significance (Supplementary Figure 3). These data do not allow us to make conclusions concerning the presence of systemic immune responses to the exposures.

### AS-expressing *C*. *elegans* fed with curli-producing bacteria displayed increased AS aggregates

**Figure 3:**
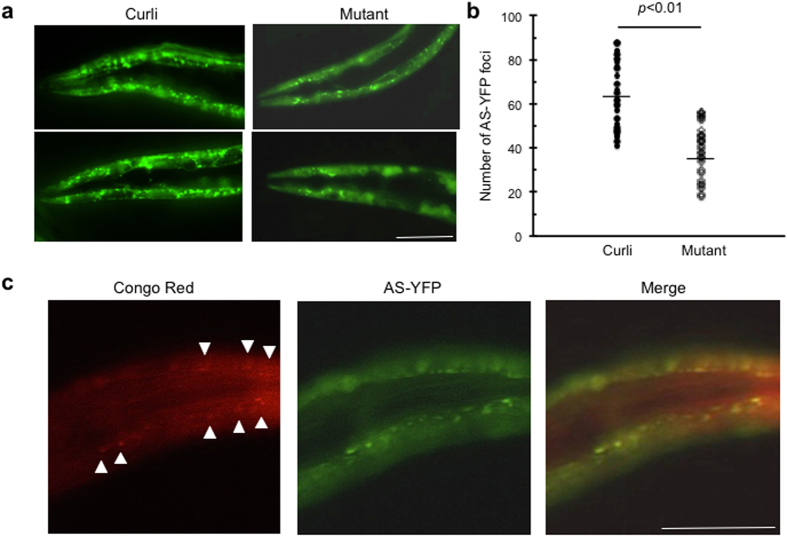
*C*. *elegans* exposed to *E*. *coli* expressing curli has enhanced AS aggregation. (**a**) Fluorescence microscopy of the anterior region of *C*. *elegans* expressing AS-YFP in the body wall muscle. Transgenic nematodes (Punc-54::AS::YFP) were age-synchronized and were fed for three days on lawns of either curli-producing *E*. *coli* or its mutant strain unable to express curli (non-curli mutant). Fluorescence microscopy was performed on immobilized live nematodes and imaging was acquired under identical conditions for the nematode groups fed with different bacterial strains. *C*. *elegans* exposed to curli expressing bacteria (Curli) contained increased numbers of larger and brighter foci of AS-YFP as compared to those exposed to non-curli expressing mutant bacteria (Mutant). Scale bar = 100 μm. (**b**) Quantitative analysis of AS-YFP foci. The number of YFP foci in the head region (from the base of the second pharyngeal bulb to the nose) of *C*. *elegans* was plotted for a group of 15 animals each exposed to either *curli*-producing *E*. *coli* (Curli) or its corresponding mutant (Mutant). The horizontal lines represent the average number of AS-YFP foci. *p* < 0.01, unpaired t test. (**c**) Congo red staining of *C*. *elegans* expressing AS-YFP exposed to curli-producing *E*. *coli*. Congo red stained deposits (arrowheads) in the head region of *C*. *elegans* expressing AS-YFP in the body wall muscles colocalized with AS-YFP aggregates. Scale bar = 100 μm.

In order to evaluate the influence of bacterial amyloid on AS aggregation in another organism we exposed AS expressing *C*. *elegans* to wild-type *E coli* expressing curli and mutant *E*. *coli* lacking the ability to synthesize curli. The nematode *C*. *elegans* naturally feeds on *E*. *coli*, and protein aggregation can be studied *in vivo* thus representing an attractive model organism to study how bacterial exposures influences AS aggregation. We utilized a transgenic (Tg) *C*. *elegans* line expressing human AS fused with YFP (AS-YFP) in body wall muscle, allowing for visualization of aggregated AS-YFP in live animals using fluorescence microscopy^23^. As shown in Fig. 3a,b AS-expressing *C*. *elegans* fed with curli-producing *E*. *coli* displayed increased AS deposits compared to those fed with the mutant *E*. *coli* lacking curli production. The Congo red stained deposits coincide with those of AS-YFP, confirming that AS-YFP forms amyloidogenic aggregates recognized by Congo red (Fig. 3c). AS-aggregates accumulated in the head first and moved to the tail during adulthood. We also stained live worms with the cytosolic oxidative stress marker CellRox Red, but no difference was seen between worms exposed or not exposed to curli-producing bacteria. This suggests that enhanced AS aggregation was not due to increased oxidative stress in the cytosol. We also tested a mitochondrial oxidative stress marker MitoSox Red, but detected only weak staining in both groups. Swimming tests were also performed to evaluate locomotive activity and showed a trend toward decreased thrashing rate (~15–20%) in worms exposed to wild-type curli-producing *E*. *coli* vs. mutant *E*. *coli* lacking curli, which did not reach statistical difference (data not shown).

## Discussion

We have shown that exposure to bacteria producing a functional extracellular amyloid protein enhances aggregation of AS in brain neurons in aged rats and in muscle cells in nematodes. In gut neurons the exposure increased levels of AS in the submucosa. The mechanism of these effects may involve upregulation of protein expression, which is well known to enhance aggregation. These effects can also have resulted from cross-seeding, which has been reported between bacterial amyloids, including curli, and mammalian proteins^24^. Lee and coworkers have demonstrated that AS aggregates seed aggregation of tau^25^. PrP^Sc^ has been reported to seed AB aggregation and curli seeds aggregation of serum amyloid A^12^. However, there have been no studies on how cross-seeding by exogenous bacterial amyloid affect the propagation and aggregation of endogenous amyloid proteins in living animals.

Our finding of enhanced AS deposition without aggregation in the gut but with aggregation in the brain is of interest. Perhaps the period of exposure was too short to allow for polymerization of AS in gut neurons. It can also be that the biophysical factors involved in induction of AS aggregation and the relationship between AS expression and aggregation are different in the gut and brain (due to pH and other factors). As the bacteria were delivered orally the pathway for induction of changes in the brain can involve the autonomic nervous system, particularly the vagus and other nerves. This is the route which is believed to be responsible, at least in part, for transmission of bovine spongiform encephalopathy and related prionoses^7^. Recent studies have also suggested the involvement of the vagus nerve in PD^26^. Brain alterations can also have been induced through oral tissues and their rich innervation as well as via a hematogenous route.

*C*. *elegans* is a genetically tractable organism for elucidating the critical events mediating interactions between bacterial amyloid proteins and AS in intestinal tissues and potential propagation of AS aggregates to neurons^27^. The finding that AS aggregation in worms is enhanced by exposure to curli expressing bacteria can also involve a cross-seeding mechanism. Since nematodes naturally take up microorganisms as food, they allow for mechanistic studies of cross-seeding by different microbial amyloid proteins expressed by distinct strains during feeding and throughout their lifespan.

Our findings of microgliosis, astrogliosis and enhanced expression of IL-6, TLR2 and TNF in the brain following curli exposure suggest the occurrence of an enhanced local sterile inflammatory response to AS in the brain. These findings do not appear to be caused by T cell activation by bacterial amyloid, as cellular infiltrates were not found in the brain or other tissues. Activation of the immune system in both AD and PD have now been extensively established^28^. The importance of the immune system in AD was first proposed by Braak and colleagues^6^, and has been recently supported by the association of immune system genes involved in neurodegeneration (for recent review see Colonna and Wang^14^). It has been shown that impaired microglial proliferation slows AD in Tg mice^29^. Bacterial amyloid is recognized as a pathogen associated molecular pattern (PAMP) with messengers including TLR2/1, CD14, NFkB and iNOS^19^. These messengers of the innate immune system are also involved in its recognition of AS, AB and also curli. Recently it has been shown that TLR2 inhibition prevents AS aggregation through activation of autophagy^20^, suggesting that TLR2 activation through exposure to bacterial amyloid is pathogenic. Microglial TLR2 activation has also been reported to increase cellular uptake of AB and is involved in AB stimulated microglial activation^30^. The co-receptor molecule CD14 has also been linked to oxidative damage and dendritic degeneration following innate immune system activation^31^.

Priming of immune cells to respond to these bacterial or other amyloids in the gut may cause enhanced responses to neuronal amyloids in the brain. Gallo *et al*.^32^ have recently observed that bacterial amyloid binds to bacterial and eukaryotic DNA and causes amyloid polymerization, as well as enhancement of autoimmunity. The intensity of the immune system’s response to cerebral amyloid deposition is important in determining the development of dementia in AD and PD, as many older persons have been shown to have significant AB pathology without dementia and without microglial activation^17^.

The precise role of protein aggregation and inflammation in neurodegeneration remains unclear^33^. Aggregation of AS and production of toxic oligomers are pathogenic, but intracellular AS aggregation into fibrils can be protective^34^. Furthermore, it has been shown in studies of the molecular machinery for the production of curli, that the same protein accelerated the aggregation of one protein and inhibited the amyloid formation of another^35^. There is also evidence that an active immune response is neuroprotective, as suggested by the work on immunotherapy for AD. Immune responses participate in clearance of AB plaques and improved cognition in animal studies^36^ and microglia produce a barrier reducing the toxic effects of AB on neurotic dystrophy^37^. It has been proposed that the association of AD and PD with age is related to senescence of the immune system and lowered immune responses^38^. Thus it is conceivable that bacterial amyloids induce either a pathogenic or salutogenic effect of neurodegenerative processes in the brain^39^,^40^. Our study did not include behavioral measures and thus the effect of exposure to bacterial amyloid on the neurodegenerative phenotype resulting from the enhanced AS aggregation and immune response remains to be determined.

There is now a vast literature documenting the influence of the microbiota on metabolism, immunity, cancer, diabetes, obesity, intestinal diseases, heart disease and other conditions. Bacteria, fungi and other organisms comprising our microbiota make functional amyloid proteins. Biofilms are found in the body and up to 40% of bacterial biofilms have amyloids^9^. Evidence for a role for fungi in AD has recently been proposed, but the possible role for fungal amyloids has not yet been considered^41^. Although it is now documented that many bacteria that make amyloid proteins are components of our microbiota, the presence of these proteins in the body has not been comprehensively evaluated^32^. Although *E coli* is not a major component of our microbiota there are several other species that make amyloid proteins that are important commensal partners, including *Streptococcus mutans*, *Staphlococcus aureus*, *Salmonella enterica*, *Mycobacterium tuberculosis* and others^8^,^42^. Gene homologs encoding curli were recently determined also in four phyla: *Bacteroidetes*, *Proteobacteria*, *Firmicutes*, *and Thermodesulfobacteria*^8^. It is tempting to speculate that the various amyloids to which we are exposed influence misfolding of endogenous proteins with strain specificity, as has been reported for AS, AB and related proteins. That is, certain bacterial amyloids are particularly important for inducing aggregation of certain strains of a neurodegenerative disease protein, and this is reflected in distinct disease phenotypes^43^,^44^.

Our work suggests that protein misfolding and immune activation in neurodegenerative disorders are triggered through cross-seeding by exposure to exogenous microbial amyloids in the nose, mouth and gut. We need to elucidate the molecular mechanisms responsible for the observed effects of exposure to bacterial amyloid. Cross-seeding of amyloidogenic proteins by bacterial amyloids has been documented in both *in vivo*^12^ and *in vitro*^24^ (e.g, curli can cause cross-seeding of serum amyloid A^12^). We provide evidence for our proposed mechanism for the induction of neuroinflammation in the brain: the innate immune system utilizes TLR2 to recognize bacterial amyloid^45^, and we demonstrate upregulation of TLR2 in the striatum and hippocampus of animals exposed to bacteria producing curli (Fig. 2F). The scope of our study did not include the influence of enhanced AS aggregation on the function of the animals or the impact of bacterial amyloid on oxidative toxicity. The pathway by which oral and intestinal bacterial amyloid influence brain processes also remains to be documented. We also do not know which of the many bacterial amyloids are of particular importance. A metaproteomic analysis of the amyloid proteins present in the mouth and intestines has not been accomplished and will be of great interest^46^.

Two recent studies have also provided evidence for the potential role of intestinal bacteria in neurodegeneration. Minter and colleagues have shown that changes in the gut microbiota induced by antibiotics alter neuroinflammation and amyloid deposition in a mouse model of AD^47^. Harach *et al*. have observed that germ-free AD model mice have reduced AB pathology in the brain, compared to conventionally raised animals^48^. The possible contribution of bacterial amyloid to these findings has not been evaluated.

This is one of the first studies that evaluates the influence of bacterial amyloid on disease processes in living animals^40^. Our data suggest that amyloid proteins in the microbiota are involved in the origination and maintenance of neurodegenerative disease. Further studies on the influence of our partner organisms on the brain are certainly indicated. If the results are supported by further analysis and experimentation there are a broad range of preventive and therapeutic measures that will be of value.

### Experimental Procedures

#### Antibodies and chemicals

The protease and phosphatase inhibitor cocktails and Congo red were purchased from Sigma-Aldrich (St Louis, MO). Hypoestoxide was obtained from Immune Modulation, Inc. (Bloomington, CA). The following antibodies were used: α-synuclein (Syn-1; BD Bioscience, San Diego, CA); TNFα, glial fibrillary acidic protein (GFAP) (GA5), TH, and NeuN (Millipore, County Cork, Ireland); β-actin (Sigma-Aldrich, St Louis, MO); NF-κB p65 and phospho-NF-κB p65 (Cell Signaling, Beverly, MA); IL-1β and IL6 (Abcam, Cambridge, MA); α-synuclein (CT, Syn105) [18]; α-synuclein (syn211) (Life Technologies, Grand Island, NY); and Iba-1 (Wako, Richmond, VA).

### Animals

Aged male Fischer 344 rats were obtained from the National Institute of Aging, NIH (M. Murthy). These rats were chosen because it has been described that their neurons in the gut develop deposits of AS with age, thus mimicking PD^21^. At the time of euthanasia via CO_2_ asphyxiation the rats were 22.5–25 months of age. All animal procedures were approved by the Institutional Animal Care and Use Committee of the University of Louisville (Protocol 12022) and were also in compliance with United States Public Health Service standards and National Institutes of Health guidelines. The University of Louisville Animal Care Facility is accredited by AAALAC International.

### Bacteria preparation and exposure

A wild-type *E*. *coli* strain LSR12 and the isogenic mutant lacking both curli operons (strain C600 with deletion of the curli operons) were obtained through the generosity of M. Chapman, University of Michigan. The identity of strains has been verified using PCR with oligonucleotides priming at individual curli genes and spanning the entire curli region (oligonucleotide sequences are available upon request). We also confirmed by Congo red (CR) staining that the wild-type strain is CR-positive while curli mutant is CR-negative (data not shown). The bacteria were otherwise identical. *E*. *coli* was grown in YESCA medium (1g/l Yeast Extract, 10g/l casamimo acids) at 37 °C. TSBYE which consists of 30 grams per liter trypticase soy broth (Difco) supplemented with 2% (w/v) yeast extract, 1 mg/ml hemin (final concentration), and 5 mg/ml menadione (final concentration) (10% CO_2_, 10% H_2_, and 80% N_2_) at 37 °C for 48 hours.

Oral exposure of rats was performed essentially as previously described by Baker *et al*.^49^. A total of 11–13 rats per group were used per experiment. Animals were initially treated with sulfamethoxazole (MP Biomedical, Solon, OH) at a final concentration of 800 mg/ml and trimethoprim (Sigma, St. Louis, MO) at a final concentration of 400 mg/ml *ad libitum* for 10 days. Four days after the last antibiotic treatment, the rats were infected orally with bacteria (10^9^ cfu) suspended in 1ml 2% carboxymethylcellulsoe (CMP; MP Biomedicsal, Solon, Oh) in sterile PBS using a 2.25 feeding needle (Popper and Sons, Inc. New Hyde Park NY). Animals were inoculated orally every week for 2 months (N = 19) or 3 months (N = 14). Animals were studied in 3 groups: Vehicle only (N = 13); exposure to wild type bacteria (curli-producing) (N = 11); and exposure to mutant bacteria (lacking curli operon) (N = 9). The vehicle control group was treated with CMC without bacteria. Rats were weighed several times each month. At the end of 2–3 months rats were sacrificed and various organs and blood were collected and banked.

### Serum cytokine analyses

Blood was collected from the hearts of the aged rats treated with or without curli-producing *E*. *coli*, and control rats without any treatments (N = 7–8 per group). The blood samples were centrifuged at 4 °C (1,000 × g) for 30 min to extract the serum. The sera were stored in aliquots at −80 °C until use. ELISAs were performed according to the manufacturer’s protocol. Serums IL-1β, IL-6, IL-10, INF-γ, and TNF-α were measured by R&D Systems Quantikine ELISA kits and serum TLR-2 was determined by an ELISA kit from Antibodies-Online (Atlanta, Georgia). Data were expressed as optical density (O.D.) value at 450 nm with double wells per sample and the wavelength correction was set at 570 nm. A standard curve was created for each tested cytokine at each time to determine the concentration of the target cytokine concentration in each sample. The concentration of the positive control sample was within the linear range of the standard curve. Results presented as means ± SE, and statistical analysis was done using GraphPad Prism software. One-way analysis of variance (ANOVA) with Turkey post-hoc test was used for multiple comparisons among groups. p < 0.05 was considered statistically significant. Positive controls were tested to confirm the function of the assays.

### Immunocytochemistry

The neuropathological studies were carried out on blinded specimens. The procedures for immunohistochemical, immunofluorescence, and neuropathological analysis have been described elsewhere^50^. Briefly, the right hemibrains were post-fixed in phosphate-buffered 4% paraformaldehyde at 4 °C for neuropathological analysis, blind-coded sagittal brain sections were incubated with primary antibodies at 4 °C for overnight following serial sectioning in the sagittal plane at 40 μm with a Vibratome 2000 (Leica, Deerfield, IL) for neuropathological and immunocytochemical studies. The next day, sections were incubated with either biotinylated- or FITC-conjugated secondary antibodies and detected with avidin D-HRP HRP (ABC elite, Vector Laboratories, Burlingame, CA) and with Tyramide Signal Amplification Direct system (PerkinElmer, Waltham, MA), respectively. Analysis of AS accumulation was performed using free-floating, blind-coded sections^51^. To determine the neuroinflammation, neurodegeneration, accumulation of α-synuclein, and NF-κB activation, we stained brain sections with Iba-1, GFAP, TNFα, IL- 1β, IL6, human α-synuclein, NF-κB, and phosphorylated NF-κB antibodies, respectively. Sections were imaged by Olympus BX41 microscope. All immunoreactivity levels were determined by optical density analysis using Image Quant 1.43 program (NIH) except the immunoreactivity of Iba-1. The cell numbers of Iba-1-positive cells were determined per field (230 μmÅ ~ 184 μm) of each animal based on cell body recognition using Image Quant 1.43 program (NIH). Immunohistochemistry was performed with antibody against full length AS, before and after proteinase K exposure. Sections were incubated overnight at 4 °C with antibodies against total a-syn (1:500, affinity purified rabbit polyclonal, Millipore)^50^, GFAP (mouse monoclonal, Millipore), Iba-1 (mouse monoclonal, Wako laboratories), IL6 (mouse monoclonal Cell Signaling), TLR2 (mouse monoclonal Millipore), TNFa (mouse monoclonal Cell Signaling), IL1beta (mouse monoclonal Abcam) followed by biotin-tagged anti-rabbit or anti-mouse IgG1 (1:100, Vector Laboratories, Inc., Burlingame, CA) secondary antibodies, Avidin D-HRP (1:200, ABC Elite, Vector), and visualized with diaminobenzidine. Sections were scanned with a digital Olympus bright field digital microscope (BX41).

### Caenorhabditis elegans studies

Standard conditions were used for *C*. *elegans* propagation on NGM (nematode growth medium) plates seeded with *E*. *coli* OP50-1 at 20 °C. Transgenic (Tg) *C*. *elegans* line expressing human AS fused with YFP (AS-YFP) (strain NL5901, Punc-54::AS::YFP) in body wall muscle^23^ was obtained from the Caenorhabditis Genetics Center (CGC). The use of these animals allowed for visualization of aggregated AS-YFP in live animals using fluorescence microscopy. Tg AS-YFP nematodes were age-synchronized by hypochlorite bleaching, hatched overnight and were subsequently cultured on NGM plates seeded with *E*. *coli* OP50-1 until larval stage L4 (adult day 0). They were then fed for three days solely on fresh NGM plates seeded with either curli-producing *E*. *coli* or mutant *E*. *coli* unable to produce curli (non-curli mutant). Fluorescence microscopy was performed on immobilized live animals to visualize AS-YFP using an eVOS microscope (Life Technologies) and imaging was acquired under identical conditions for nematodes fed with either wild-type or mutant bacteria. The experiments were repeated three times, each with 10 worms for each treatment group. As characterized previously^23^, AS aggregates are recognized as fluorescent foci of inclusions containing AS-YFP within muscle cells in this *C*. *elegans* model.

Congo red staining of worms was performed using the procedures modified from that described previously^52^. Worms were fixed by incubation in 4% paraformaldehyde in Dulbecco’s phosphate buffered saline for 15 h at 4 °C, and were permealized at 37 °C for 15 h in a solution of 1% Triton X-100, 5% beta-mercaptoethanol, 125 mM Tris-HCl, pH 7.5. Worms were then mounted on glass slide and stained with 0.5 mg/ml Congo red in 50% ethanol for 1 min. Destaining was immediately carried out using several rinses of 50% ethanol until solution became colorless, and then one rinse each with 75% ethanol, 50% ethanol, and water. A drop of Fluoromount was applied and stained worms were visualized for red fluorescence using a Leica fluorescence microscope.

### Statistical analysis

GraphPad Prism (GraphPad Software, San Diego, CA) was used for statistical analysis. All data are presented as means ± s.e.m and were analyzed for statistical significance by using either unpaired t test or two-way ANOVA (general linear model) followed by Bonferroni’s multiple comparison post-test. Ordinal logistic regression was used to compare the OR for gut AS deposition^53^. The statistical software package R was also used. One-way ANOVA poshoc Dunnet p < 0.05 was used when comparing to control.

## Additional Information

**How to cite this article**: Chen, S. G. *et al*. Exposure to the Functional Bacterial Amyloid Protein Curli Enhances Alpha-Synuclein Aggregation in Aged Fischer 344 Rats and *Caenorhabditis elegans*. *Sci. Rep*. **6**, 34477; doi: 10.1038/srep34477 (2016).

## Supplementary Material

Supplementary Information

## References

[b1] Walker L. C. & Jucker M. Amyloid by default. Nature neuroscience 14, 669–670 (2011). 21613991 10.1038/nn.2853 PMC10715806

[b2] Desplats P. . Inclusion formation and neuronal cell death through neuron-to-neuron transmission of alpha-synuclein. Proceedings of the National Academy of Sciences of the United States of America 106, 13010–13015 (2009). 19651612 10.1073/pnas.0903691106 PMC2722313

[b3] Prusiner S. B. Cell biology. A unifying role for prions in neurodegenerative diseases. Science 336, 1511–1513 (2012). 22723400 10.1126/science.1222951 PMC3942086

[b4] Friedland R. P. Mechanisms of molecular mimicry involving the microbiota in neurodegeneration. Journal of Alzheimer’s disease: JAD 45, 349–362 (2015). 25589730 10.3233/JAD-142841

[b5] Soto C. Transmissible proteins: expanding the prion heresy. Cell 149, 968–977 (2012). 22632966 10.1016/j.cell.2012.05.007 PMC3367461

[b6] Braak H. . Staging of brain pathology related to sporadic Parkinson’s disease. Neurobiology of aging 24, 197–211 (2003). 12498954 10.1016/s0197-4580(02)00065-9

[b7] van Keulen L. J., Vromans M. E. & van Zijderveld F. G. Early and late pathogenesis of natural scrapie infection in sheep. APMIS: acta pathologica, microbiologica, et immunologica Scandinavica 110, 23–32 (2002). 10.1034/j.1600-0463.2002.100104.x 12064252

[b8] Hufnagel D. A., Tukel C. & Chapman M. R. Disease to dirt: the biology of microbial amyloids. PLoS pathogens 9, e1003740 (2013). 24278013 10.1371/journal.ppat.1003740 PMC3836715

[b9] Larsen P. . Amyloid adhesins are abundant in natural biofilms. Environ Microbiol 9, 3077–3090 (2007). 17991035 10.1111/j.1462-2920.2007.01418.x

[b10] Cherny I. . The formation of Escherichia coli curli amyloid fibrils is mediated by prion-like peptide repeats. Journal of molecular biology 352, 245–252 (2005). 16083908 10.1016/j.jmb.2005.07.028

[b11] Evans M. L. . The bacterial curli system possesses a potent and selective inhibitor of amyloid formation. Mol Cell 57, 445–455 (2015). 25620560 10.1016/j.molcel.2014.12.025 PMC4320674

[b12] Lundmark K., Westermark G. T., Olsen A. & Westermark P. Protein fibrils in nature can enhance amyloid protein A amyloidosis in mice: Cross-seeding as a disease mechanism. Proceedings of the National Academy of Sciences of the United States of America 102, 6098–6102 (2005). 15829582 10.1073/pnas.0501814102 PMC1087940

[b13] Solomon A. . Amyloidogenic potential of foie gras. Proceedings of the National Academy of Sciences of the United States of America 104, 10998–11001 (2007). 17578924 10.1073/pnas.0700848104 PMC1894569

[b14] Colonna M. & Wang Y. TREM2 variants: new keys to decipher Alzheimer disease pathogenesis. Nat Rev Neurosci 17, 201–207 (2016). 26911435 10.1038/nrn.2016.7

[b15] Zenaro E. . Neutrophils promote Alzheimer’s disease-like pathology and cognitive decline via LFA-1 integrin. Nature medicine 21, 880–886 (2015). 10.1038/nm.3913 26214837

[b16] Holmes C. Review: systemic inflammation and Alzheimer’s disease. Neuropathology and applied neurobiology 39, 51–68 (2013). 23046210 10.1111/j.1365-2990.2012.01307.x

[b17] Akiyama H. . Inflammation and Alzheimer’s disease. Neurobiology of aging 21, 383–421 (2000). 10858586 10.1016/s0197-4580(00)00124-x PMC3887148

[b18] Lathrop S. K. . Peripheral education of the immune system by colonic commensal microbiota. Nature 478, 250–254 (2011). 21937990 10.1038/nature10434 PMC3192908

[b19] Tukel C. . Toll-like receptors 1 and 2 cooperatively mediate immune responses to curli, a common amyloid from enterobacterial biofilms. Cellular microbiology 12, 1495–1505 (2010). 20497180 10.1111/j.1462-5822.2010.01485.x PMC3869100

[b20] Kim C. . Antagonizing Neuronal Toll-like Receptor 2 Prevents Synucleinopathy by Activating Autophagy. Cell Rep 13, 771–782 (2015). 26489461 10.1016/j.celrep.2015.09.044 PMC4752835

[b21] Phillips R. J., Walter G. C., Ringer B. E., Higgs K. M. & Powley T. L. Alpha-synuclein immunopositive aggregates in the myenteric plexus of the aging Fischer 344 rat. Experimental neurology 220, 109–119 (2009). 19664623 10.1016/j.expneurol.2009.07.025 PMC2761519

[b22] Baker P. J., Dixon M. & Roopenian D. C. Genetic control of susceptibility to Porphyromonas gingivalis-induced alveolar bone loss in mice. Infection and immunity 68, 5864–5868 (2000). 10992496 10.1128/iai.68.10.5864-5868.2000 PMC101548

[b23] van Ham T. J. . C. elegans model identifies genetic modifiers of alpha-synuclein inclusion formation during aging. PLoS genetics 4, e1000027 (2008). 18369446 10.1371/journal.pgen.1000027 PMC2265412

[b24] Hartman K. . Bacterial curli protein promotes the conversion of PAP248-286 into the amyloid SEVI: cross-seeding of dissimilar amyloid sequences. PeerJ 1, e5 (2013). 23638387 10.7717/peerj.5 PMC3629062

[b25] Guo J. L. & Lee V. M. Seeding of normal Tau by pathological Tau conformers drives pathogenesis of Alzheimer-like tangles. The Journal of biological chemistry 286, 15317–15331 (2011). 21372138 10.1074/jbc.M110.209296 PMC3083182

[b26] Klingelhoefer L. & Reichmann H. Pathogenesis of Parkinson disease–the gut-brain axis and environmental factors. Nature reviews. Neurology 11, 625–636 (2015). 26503923 10.1038/nrneurol.2015.197

[b27] Nussbaum-Krammer C. I. & Morimoto R. I. Caenorhabditis elegans as a model system for studying non-cell-autonomous mechanisms in protein-misfolding diseases. Dis Model Mech 7, 31–39 (2014). 24396152 10.1242/dmm.013011 PMC3882046

[b28] Allen Reish H. E. & Standaert D. G. Role of alpha-synuclein in inducing innate and adaptive immunity in Parkinson disease. J Parkinsons Dis 5, 1–19 (2015). 25588354 10.3233/JPD-140491 PMC4405142

[b29] Olmos-Alonso A. . Pharmacological targeting of CSF1R inhibits microglial proliferation and prevents the progression of Alzheimer’s-like pathology. Brain: a journal of neurology 139, 891–907 (2016). 26747862 10.1093/brain/awv379 PMC4766375

[b30] Reed-Geaghan E. G., Savage J. C., Hise A. G. & Landreth G. E. CD14 and toll-like receptors 2 and 4 are required for fibrillar A{beta}-stimulated microglial activation. The Journal of neuroscience: the official journal of the Society for Neuroscience 29, 11982–11992 (2009). 19776284 10.1523/JNEUROSCI.3158-09.2009 PMC2778845

[b31] Milatovic D., Zaja-Milatovic S., Montine K. S., Shie F. S. & Montine T. J. Neuronal oxidative damage and dendritic degeneration following activation of CD14-dependent innate immune response *in vivo*. Journal of neuroinflammation 1, 20 (2004). 15498098 10.1186/1742-2094-1-20 PMC527876

[b32] Gallo P. M. . Amyloid-DNA Composites of Bacterial Biofilms Stimulate Autoimmunity. Immunity 42, 1171–1184 (2015). 26084027 10.1016/j.immuni.2015.06.002 PMC4500125

[b33] Selkoe D. J. & Hardy J. The amyloid hypothesis of Alzheimer’s disease at 25 years. EMBO Mol Med (2016). 10.15252/emmm.201606210 PMC4888851 27025652

[b34] Poehler A. M. . Autophagy modulates SNCA/alpha-synuclein release, thereby generating a hostile microenvironment. Autophagy 10, 2171–2192 (2014). 25484190 10.4161/auto.36436 PMC4502760

[b35] Chorell E. . Bacterial Chaperones CsgE and CsgC Differentially Modulate Human alpha-Synuclein Amyloid Formation via Transient Contacts. PloS One 10, e0140194 (2015). 26465894 10.1371/journal.pone.0140194 PMC4605646

[b36] De Strooper B. & Karran E. The Cellular Phase of Alzheimer’s Disease. Cell 164, 603–615 (2016). 26871627 10.1016/j.cell.2015.12.056

[b37] Condello C., Yuan P., Schain A. & Grutzendler J. Microglia constitute a barrier that prevents neurotoxic protofibrillar Abeta42 hotspots around plaques. Nature communications 6, 6176 (2015). 10.1038/ncomms7176 PMC4311408 25630253

[b38] Chen A. . Multiplex analyte assays to characterize different dementias: brain inflammatory cytokines in poststroke and other dementias. Neurobiology of aging 38, 56–67 (2016). 26827643 10.1016/j.neurobiolaging.2015.10.021 PMC4759608

[b39] Raby A. C. . Soluble TLR2 reduces inflammation without compromising bacterial clearance by disrupting TLR2 triggering. J Immunol 183, 506–517 (2009). 19542461 10.4049/jimmunol.0802909

[b40] Oppong G. O. . Biofilm-associated bacterial amyloids dampen inflammation in the gut: oral treatment with curli fibres reduces the severity of hapten-induced colitis in mice. NPJ Biofilms Microbiomes 1 (2015). 10.1038/npjbiofilms.2015.19 PMC4739805 26855788

[b41] Gilchrist K. B., Garcia M. C., Sobonya R., Lipke P. N. & Klotz S. A. New features of invasive candidiasis in humans: amyloid formation by fungi and deposition of serum amyloid P component by the host. J Infect Dis 206, 1473–1478 (2012). 22802434 10.1093/infdis/jis464 PMC3570177

[b42] Schwartz K. & Boles B. R. Microbial amyloids–functions and interactions within the host. Current opinion in microbiology 16, 93–99 (2013). 23313395 10.1016/j.mib.2012.12.001 PMC3622111

[b43] Aguzzi A. Neurodegeneration: Alzheimer’s disease under strain. Nature 512, 32–34 (2014). 25100477 10.1038/512032a

[b44] Peelaerts W. . alpha-Synuclein strains cause distinct synucleinopathies after local and systemic administration. Nature 522, 340–344 (2015). 26061766 10.1038/nature14547

[b45] Rapsinski G. J. . Toll-like receptor 2 and NLRP3 cooperate to recognize a functional bacterial amyloid, curli. Infect Immun 83, 693–701 (2015). 25422268 10.1128/IAI.02370-14 PMC4294241

[b46] Wilmes P., Heintz-Buschart A. & Bond P. L. A decade of metaproteomics: where we stand and what the future holds. Proteomics 15, 3409–3417 (2015). 26315987 10.1002/pmic.201500183 PMC5049639

[b47] Minter M. R. . Antibiotic-induced perturbations in gut microbial diversity influences neuro-inflammation and amyloidosis in a murine model of Alzheimer’s disease. Sci Rep. 21, 6:30028 (2016). 10.1038/srep30028 PMC4956742 27443609

[b48] Harach T. . Reduction of Alzheimer’s disease beta-amyloid pathology in the absence of gut microbiota arXiv:1509.02273[q-bio.MN] (2015).

[b49] Baker P. J., DuFour L., Dixon M. & Roopenian D. C. Adhesion molecule deficiencies increase Porphyromonas gingivalis-induced alveolar bone loss in mice. Infection and immunity 68, 3103–3107 (2000). 10816450 10.1128/iai.68.6.3103-3107.2000 PMC97538

[b50] Hsu L. J. . Alpha-synuclein promotes mitochondrial deficit and oxidative stress. Am J Pathol. 157, 401–10 (2000). 10934145 10.1016/s0002-9440(10)64553-1 PMC1850140

[b51] Hsu L. J., Jan M. S. & Lin Y. S. *In vivo* staphylococcal enterotoxin B (SEB)-primed murine splenocytes secrete mediators which suppress CD25(hi) expression and cell cycle progression of naive splenocytes in response to SEB *in vitro*. Cell Immunol 201, 50–57 (2000). 10805973 10.1006/cimm.2000.1628

[b52] Wu Y. . Amyloid-beta-induced pathological behaviors are suppressed by Ginkgo biloba extract EGb 761 and ginkgolides in transgenic Caenorhabditis elegans. The Journal of neuroscience: the official journal of the Society for Neuroscience 26, 13102–13113 (2006). 17167099 10.1523/JNEUROSCI.3448-06.2006 PMC6674971

[b53] McCullagh P. Regression Models for Ordinal Data. J. R. Statist. Soc. 42, 109–142 (1980).

