# Supplementary material for: Exposure to the Functional Bacterial Amyloid Protein Curli Enhances Alpha-Synuclein Aggregation in Aged Fischer 344 Rats and Caenorhabditis elegans
Source: Sci Rep. 2016 Oct 6;6:34477. doi: 10.1038/srep34477 (PMC5052651; doi:10.1038/srep34477)
Supplement: Supplementary Information [file srep34477-s1.pdf]

Supplementary material

Exposure to the Functional Bacterial Amyloid Protein Curli Enhances Alpha-Synuclein Aggregation in Aged Fischer 344 Rats and *Caenorhabditis elegans*

Shu G. Chen, PhD (1)

Vilius Stribinskis, PhD (2)

Madhavi J. Rane, PhD (3)

Donald Demuth, PhD (4)

Evelyne Gozal, PhD (5)

Andrew M. Roberts, PhD (6)

Rekha Jagadapillai, PhD (5)

Ruolan Liu, PhD (7)

Kyonghwan Choe (1)

Bhooma Shivakumar (1)

Francheska Son (1)

Shinying Jin, PhD (3)

Richard Kerber PhD (9)

Anthony Adame (8)

Eliezer Masliah, PhD, MD (8)

Robert P. Friedland, MD* (7)

1. Dept. of Pathology, Case Western Reserve University, Cleveland, Ohio
2. Brown Cancer Center, University of Louisville School of Medicine, Louisville, Kentucky
3. Dept. of Medicine, University of Louisville School of Medicine, Louisville, Kentucky
4. Dept. of Oral Immunology and Infectious Diseases, University of Louisville School of Dentistry, Louisville, Kentucky
5. Dept. of Pediatrics, University of Louisville School of Medicine, Louisville, Kentucky
6. Dept. of Physiology, University of Louisville School of Medicine, Louisville, Kentucky
7. Dept. of Neurology, University of Louisville School of Medicine, Louisville, Kentucky
8. Laboratory of Experimental Neuropathology, University of California San Diego
9. Dept. of Epidemiology and Population Health, University of Louisville School of Public Health, Louisville, Kentucky

* Author for correspondence: Robert P. Friedland, MD, Rudd Chair and Professor, Department of Neurology, HSC A119, 550 South Preston Rd, Louisville 502 896-1446; 502 896 6344 FAX [robert.friedland@louisville.edu]

Supplementary material

Cytokine ELISA assays

Serum samples were collected from 20-21 month-old male Fisher (F344) rats treated with or without curli-producing *E. Coli*, as well as control rats without exposure to bacteria (N = 7-8 per group). Following the manufacturer's instruction, serum IL-1β, IL-6, IL-10, INF-γ, and TNF-α were measured by R&D System Quantikine ELISA kits and serum TLR-2 was determined by an ELISA kit from Antibodies-Online (Atlanta Georgia). Data were expressed as optical density (O.D.) value at 450 nm with double wells per sample and the wavelength correction was set at 570 nm. A standard curve was created for each tested cytokine at each time to determine the concentration of the target cytokine concentration in each sample. The concentration of the positive control sample was within the linear range of the standard curve. Results presented as means ± SE, and statistical analysis was done using GraphPad Prism software. One-way analysis of variance (ANOVA) with Turkey post-hoc test was used for multiple comparisons among groups. $p < 0.05$ was considered statistically significant.

Cytokine IL-1β, IL-6, IL-10, TNF-α, and INF-γ

Serum INF-γ was detected from all three groups of rats (Supplementary Figure 3A). The levels of INF-γ were not significantly different among the three groups. (Treated with curli-producing *E. Coli* group: 31.8±14.2 pg/ml, Treated without curli-producing *E. Coli* group: 15.5±7.8 pg/ml, control group: 55.3±24.1 pg/ml). The serum levels of IL-1β, IL-6, IL-10, and TNF-α were low and could not be detected. See Supplementary Figure 3A.

Supplementary Figures

Supplementary Figure 1

Standard Histology: Plastic Sections

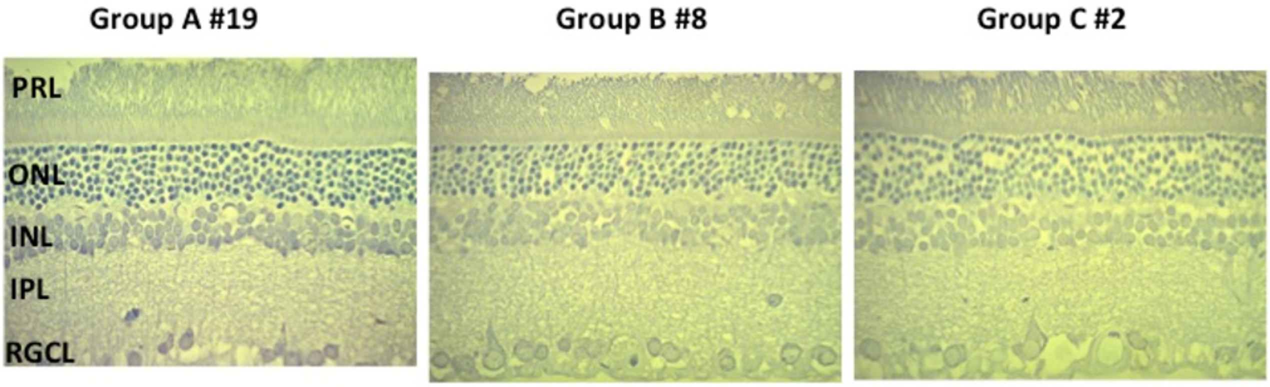

The laminar organization of the retina appears similar in all groups. INL: inner nuclear layer, IPL: inner plexiform layer, ONL: outer nuclear layer, PRL: photoreceptor layer, RGCL: retinal ganglion cell layer.

Supplementary Figure 1

Plastic sections of retina from Group A (mutant *E coli* lacking the curli operons); Group B (wild type *E coli* producing curli); and Group C (control receiving only vehicle). Differences between the groups were not observed.

Supplementary Figure 2

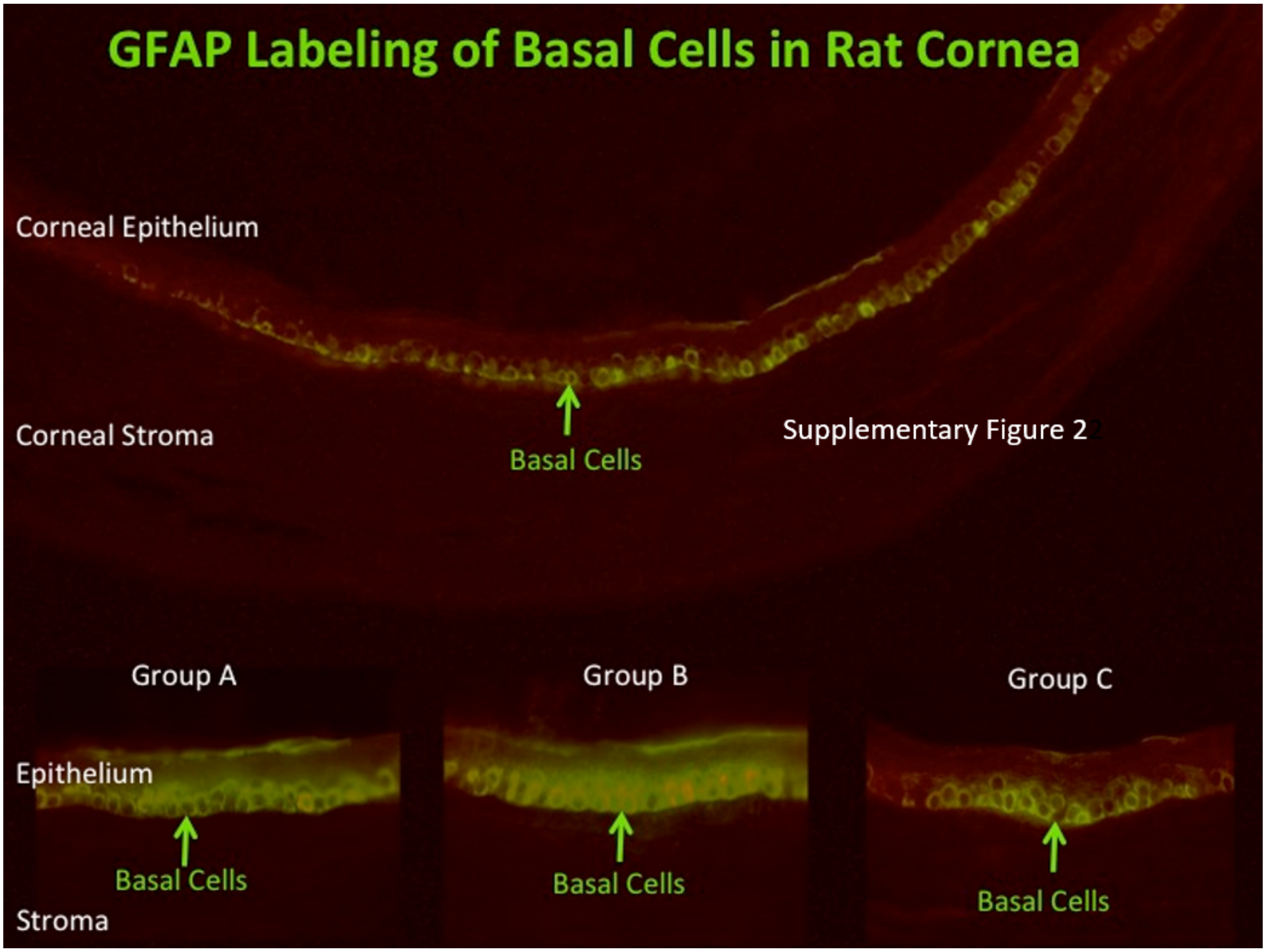

GFAP labeling of basal cells in rat cornea. Groups as described for Figure 1. Differences between the groups was not observed.

Supplementary Figure 3

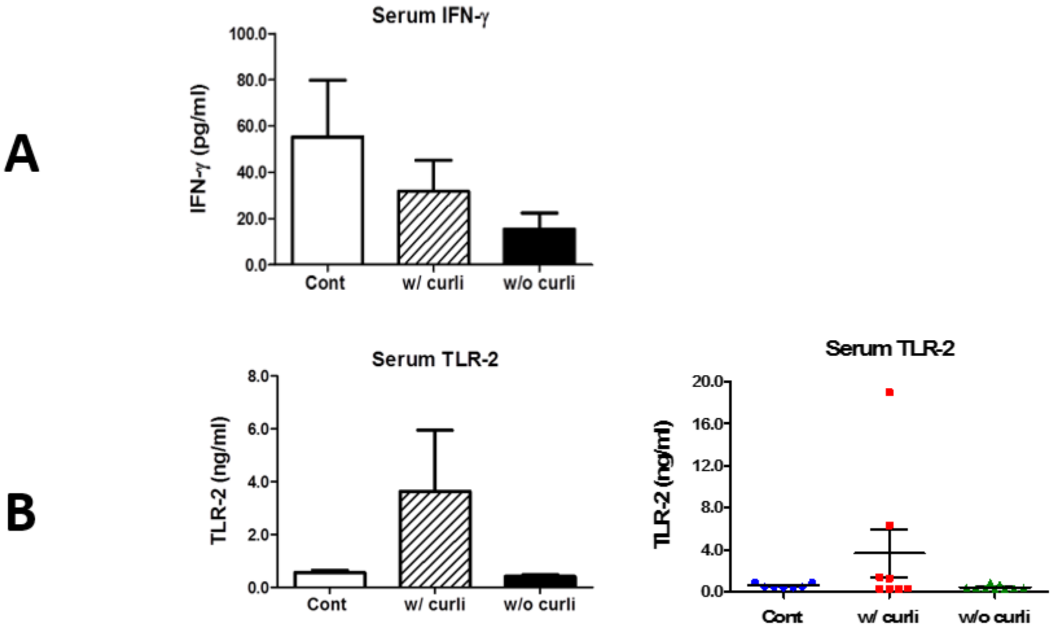

Supplementary Figure 3

Serum levels of IFN-g and TLR-2

Sera were obtained from Control animals (cont, N = 7), animals exposed to curli-producing wild type bacteria (w/ curli, N = 8), and animals exposed to bacteria unable to produce curli (w/o curli, N = 9). Serum IFN-g (A) and TLR-2 (B) were measured by ELISA assay. Data is expressed as O.D. value (mean ± SE). All data are representative of two assays with similar results. There were no significant differences detected.

Suppl. Fig 4

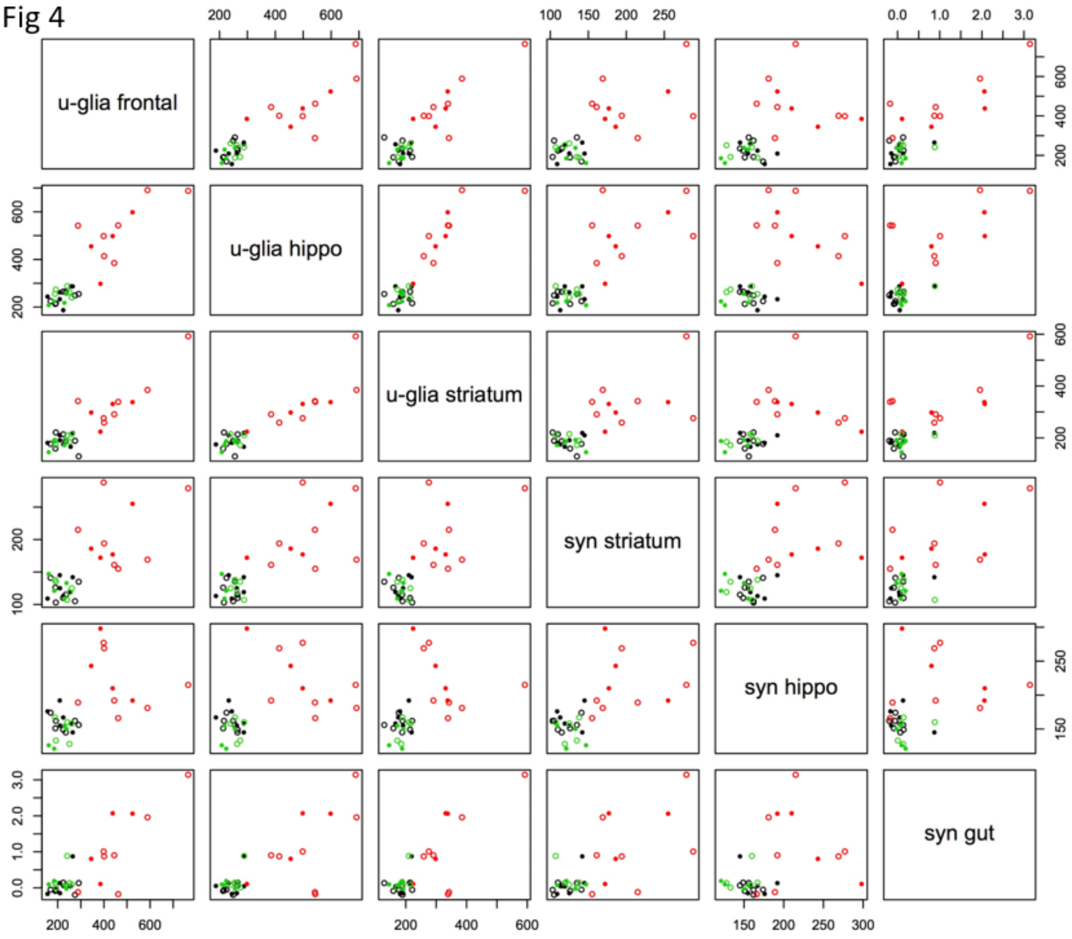

Scatterplot matrix of microglial cell counts and synuclein optical densities for the two subgroups of unexposed (A-black and C-green) animals compared to exposed animals (B-red). Open and closed circles represent the first and second experimental replicates, respectively. For each measured variable, the exposed animals exhibited substantially greater evidence of inflammation than the two groups of unexposed animals. As for the scatterplot matrix, the labels down the diagonal indicate what the axes represent. Each column has the same variable on the x-axis and each row has the same variable on the y-axis. For example, the scatterplot in the third column of the second row has "u-glia frontal" on the y-axis and "u-glia hippo" on the x-axis. The different colors also represent the three groups, showing clear differences between group B (in red) and the others, while the filled vs unfilled circles distinguish subgroups 1 and 2 (which show no difference in any group).
